# The role of maxillary sinus puncture on the diagnosis and treatment of patients with hospital-acquired rhinosinusitis

**DOI:** 10.1590/S1808-86942012000400008

**Published:** 2015-10-20

**Authors:** José Arruda Mendes Neto, Viviane Maria Guerreiro, Elcio Roldan Hirai, Eduardo Macoto Kosugi, Rodrigo de Paula Santos, Luis Carlos Gregório

**Affiliations:** MD, ENT (MSc student in the ENT graduate program at UNIFESP-EPM); MD (ENT Resident at UNIFESP-EPM); MD, ENT (Rhinology fellow at UNIFESP-EPM); MSc in ENT at UNIFESP-EPM (PhD student in the ENT graduate program at UNIFESP-EPM); PhD in ENT at UNIFESP-EPM (Assisting Physician in the course of Rhinology at UNIFESP-EPM); Associate Professor at UNIFESP-EPM (Head of the Rhinology Department at UNIFESP-EPM). UNIFESP-EPM

**Keywords:** cross infection, maxillary sinus, respiratory tract infections, sinusitis

## Abstract

Rhinosinusitis is one of the most commom causes of fever of unknown origin in critically ill patients and should be systematically searched.

**Objective**: This study aims to evaluate the diagnostic and therapeutic effect of maxillary sinus puncture performed at the bedside in patients with infective rhinosinusitis hospitalized in an Intensive Care Unit of a high complexity care hospital.

**Materials and Methods**: This retrospective study looks into patients on mechanical ventilation with fever of unknown origin and signs of rhinosinusitis on CT images who were submitted to inferior meatus maxillary sinus puncture.

**Results**: The total study sample consisted of 27 patients (70.3% male; mean age 45.3 years). The most common Intensive Care Unit admission diagnoses were head trauma and stroke. CT scans revealed the maxillary (85.2%) and sphenoid (74.1%) sinuses were the most involved paranasal sinuses. Middle meatus purulent drainage was seen in 30.7% of the nasal cavities. Fever was reduced in 70.4% of the patients after puncture (*p* < 0.001). The most commonly found organisms in sinus aspirates were *Pseudomonas aeruginosa and Acinetobacter baumannii*.

**Conclusion**: Maxillary sinus puncture performed at the bedside of the patients is an important diagnostic and theraupetic tool for critically ill patients.

## INTRODUCTION

Hospital infection (HI) is defined as an infection related to hospitalization or medical procedures carried out in a hospital setting acquired by the patient after admission that manifests during hospitalization or up to 72 hours after discharge^1^.

Studies indicate that the most common type of ICU HI is ventilator-associated pneumonia (VAP). Other frequent causes of HI are urinary tract infections associated to the use of indwelling bladder catheter and catheter-related bloodstream infections. These three causes account for over 80% of ICU HI cases^2^, ^3^, ^4^.

Infective rhinosinusitis (IRS) is usually not mentioned in large international and national studies on HI, and remains as a topic discussed only in epidemiology studies on infections on severe patients^2^, ^3^, ^5^. Nonetheless, several papers have described IRS as one of the main causes of fever in ICUs^2^, ^6^, ^7^, ^8^.

The diagnosis of IRS in ICU patients can be challenging for otorhinolaryngologists. In this group of sedated immune-depressed patients, the classic signs of rhinosinusitis are mostly absent^9^, ^10^. Clinical findings are nonspecific, and patients have fever and are often diagnosed with leukocytosis^3^, ^8^, ^11^, ^12^. Once other possible foci of infection are ruled out, ENTs are called to assess these critical patients with fever of unknown origin (FUO), leukocytosis, and signs of disorder on imaging tests.

Paranasal sinus CT scans and nasal endoscopy are the most important noninvasive tests used to assess ICU patients. Presence of air-fluid level, complete sinus opacification or mucosal thickening greater than 6 mm combined with purulent secretion in the middle meatus visualized during endoscopic examination are deemed as important signs in the diagnosis of sinusitis^13^.

Patients with FUO and radiologic signs of rhinosinusitis are first advised to remove all nasal devices^6^, ^14^, ^15^, ^16^. Additionally, topical nasal vasoconstrictors are administered for at least 72 hours before they are offered an invasive procedure^17^.

If the patient fails to respond to these initial measures, it is recommended that the infected secretion be drained from the paranasal sinus^8^, ^12^, ^14^, ^15^. This procedure offers the possibility of confirming the diagnosis of IRS and improving the patient's overall condition.

Maxillary sinus puncture is considered the gold standard to diagnose IRS in ICU patients^8^, ^12^, ^14^, ^15^. Besides offering clinical samples used to identify the causing agent of infective syndromes, it may also serve as an important tool in the treatment of this group of patients^6^, ^12^, ^15^. Authors have described success rates of approximately 70% in managing fever after removing infected secretion from the maxillary sinus^14^. The great advantage of this procedure is that it can be performed at the bedside in the ICU, thus reducing the risk of adverse effects connected to taking the patients to the OR^18^.

Critical rhinosinusitis patients with fever refractory to the procedure are traditionally referred to functional endoscopic sinus surgery. They are given general anesthesia to allow for the removal of all infected secretion, excision of local inflammatory tissue, and ventilation of the involved paranasal sinuses^16^, ^17^, ^19^.

However, ICU patients are at a higher risk for surgery, as they are immunocompromised and unstable from the hemodynamic and metabolic standpoints. Additionally, these individuals often present relevant coagulation disorders. Therefore, surgery should only be offered as a last resort for these patients.

This study aims to assess the role of maxillary sinus puncture at the bedside in the diagnosis and treatment of patients with infective rhinosinusitis admitted to the ICU of a high complexity university hospital.

## MATERIALS AND METHODS

This retrospective study looks into the ENT assessment of ICU patients with radiologic signs of rhinosinusitis with fever of unknown origin (axillary temperatures equal to or greater than 37.8°C) on ventilation staying at a high complexity hospital.

FOU was defined as fever not linked to identified disease or cases in which patients failed to respond despite being given proper therapy for a previously diagnosed condition, according to the protocol in effect at this service.

The patients and procedures described in this study were cared for and performed by a team of physicians made up by two ENT resident medical doctors (second and third year residents) and one ENT specialized in rhinology who led the team.

This study and the informed consent form presented to the patients were approved by the Ethics Committee of our institution and granted permit # 1158/09.

The patients enrolled in this study had to meet specific inclusion and exclusion criteria (consecutive allocation).

The inclusion criteria were:

• time of hospitalization greater than 48 hours;
• use of invasive ventilation;
• presence of fever of unknown origin (axillary temperature ≥ 37.8°C);
• maxillary sinus involvement verified in CT scans;
• fever persisting despite the administration of xylometazoline twice a day for 72 hours;
• absence of nasal devices for at least 72 hours;
• paranasal sinus puncture positive for infection (presence of purulent secretion and/or evidences of bacterial growth).

The exclusion criteria were:

• facial trauma with direct involvement of the paranasal sinuses;
• deviated septum touching the nasal lateral wall, increasing the complexity of the puncture procedure when done through the inferior meatus;
• polyps, antrochoanal polyps, and other nasal lesions.

Three tools were used to assess patient findings:

A- Nose endoscopy: the patients were examined with a 3.1 mm flexible fiberscope; presence of purulent secretion in the middle meatus was deemed as a sign suggestive of IRS.
B- Paranasal sinus CT scans: presence of complete opacification, air-fluid level, or mucosal thickening ≥ 6 mm.
C- Paranasal sinus puncture: positive diagnosis was defined when purulent secretion and/or evidence of bacterial growth were found^20^, ^21^.

**Figure 1:**
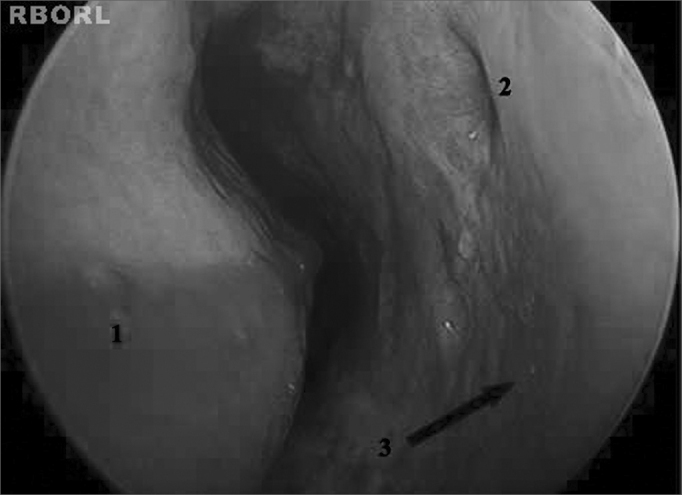
Left inferior meatus. 1: Inferior nasal concha; 2: Hasner's valve; 3: Site of puncture on the lateral wall of the left inferior meatus.

Patients underwent maxillary sinus puncture via the inferior meatus to treat IRS^7^, ^12^ (Figure 1). IRS cases refractory to puncture were treated through functional endoscopic sinus surgery (FESS); patients were placed under general anesthesia to have their involved sinuses meticulously drained^16^, ^17^, ^22^, ^23^. Proper antibiotics were administered by the intensive care team and infectologists, as verified by the analysis done on secretion aspirate cultures.

Onset of defervescence (reduction on the number of daily peaks of fever) within 48 to 72 hours^16^, ^17^ or abolition of fever within five days of the procedure^17^ where used to characterize the success of the puncture procedure. The same criteria was used to assess the success of FESS^16^, ^17^, ^22^, ^23^.

Fever abatement was used as a parameter to assess intervention effectiveness. The patients who failed to evolve favorably even after surgery were reassessed clinically and radiologically through control paranasal sinus CT scans. After discussing the case with the patient and the ICU team, a new procedure was offered whenever it was deemed necessary.

Sinus aspirates and sinus washout contents were collected and sent in sterile containers for culturing and antibiogram analysis.

The variables considered for analysis were patient gender, age, diagnosis on ICU admission, involved paranasal sinus (radiological signs), alterations on physical examination (presence or absence of purulent secretion in the middle meatus), clinical improvement after puncture, culture results, occurrence of adverse events related to the procedures, need for surgery, improved clinical outcome with surgery.

This paper used a statistical significance level of 0.05 (5%). Statistical analysis was done using the test for equality of two proportions. The test for equality of two proportions is a non-parametric test (used in low sampling) compares the proportion of answers of two variables and whether their levels are statistically significant.

Software products SPSS V16, Minitab 15 and Excel Office 2007 were used in statistical analysis.

## RESULTS

This study enrolled 27 critical patients assessed by the rhinology service physicians of a university hospital between March of 2007 and September of 2010.

**Figure 2:**
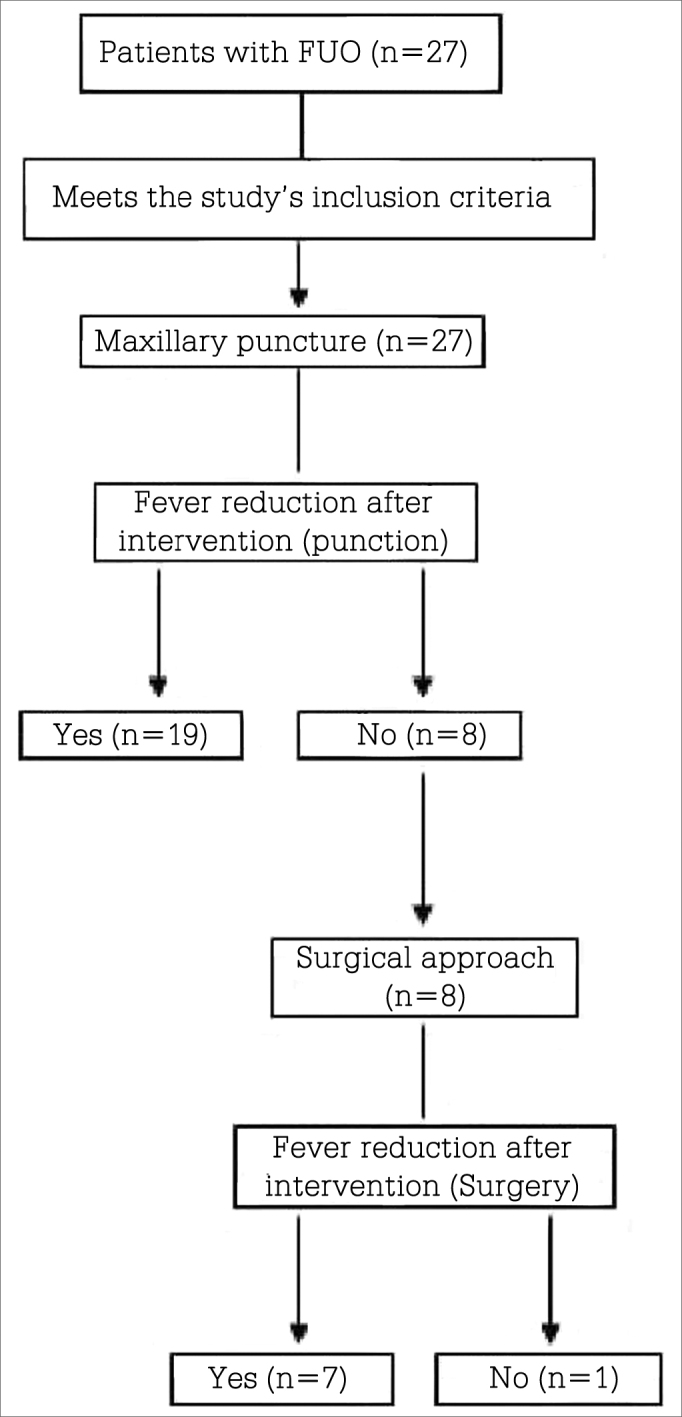
Study organizational flowchart. n = number of subjects.

Patient data can be seen on Table 1. The study flowchart is presented in Figure 2.

**Table 1:** Characteristics of the patients enrolled in the study (n = 27).

Characteristics	
Age in years*	45.3 ± 15.7
Gender	
Male	19 (70.3%)
Female	8 (29.7%)
Most frequent diagnosis at admission	
- Head trauma	11 (40.74%)
- Stroke	4 (14.8%)
- Other neurological syndromes	3 (11.1%)

n = number of subjects.

* Mean values ± standard deviation (SD).

**Figure 3:**
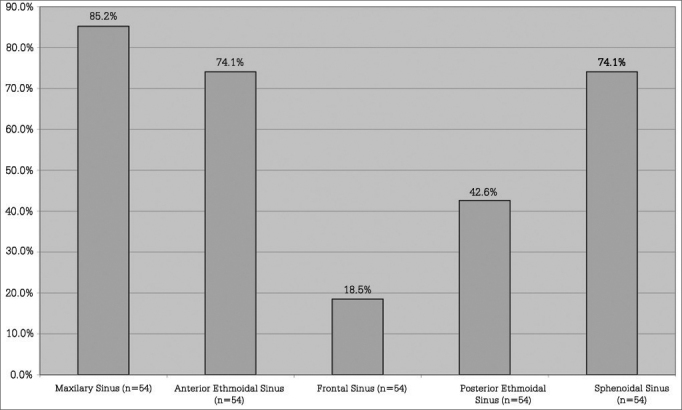
Distribution of paranasal sinuses with radiological sings of rhinosinusitis. n = number of nasal cavities.

Fifty-four nasal cavities were analyzed through CT scans. All patients had radiological signs of unilateral or bilateral maxillary and sphenoidal rhinosinusitis (Figure 3). Two patients (7.4%) had all paranasal sinuses involved as seen in CT scans.

Twenty-six nasal cavities (13 patients) were examined through endoscopy for the presence of alterations suggestive of rhinosinusitis in the middle meatus (Table 2).

**Table 2:** Distribution of nasal endoscopic examination with findings of purulent secretion in the middle meatus.

Purulent secretion	Nasal endoscopy (n = 26)	
	N	%
Middle meatus	8	30.7

n = number of subjects.

N = Number of nasal cavities with purulent secretion.

**Figure 4:**
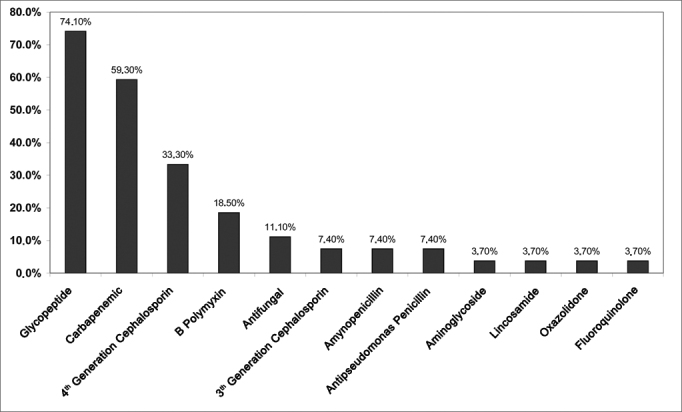
Distribution of antibiotics administered to patients diagnosed with infective rhinosinusitis.

Glycopeptide antibiotics were the most used antimicrobial agents in this study (Figure 4). The most common drug combinations were glycopeptides and carbapenems (42.6%), and glycopeptides and fourth generation cephalosporins (38.1%).

Forty-six maxillary sinus puncture procedures were performed, based on the alterations found on paranasal CT scans. Postoperative clinical improvement was observed in 70.4% of the patients (Table 3).

**Table 3:** Clinical evolution after maxillary sinus puncture.

	No		Yes		*p*-value*
Clinical improvement	n	%	n	%	
	1	12.5	7	87.5	< 0.001

n = number of subjects.

* Test for equality of two proportions.

No complications were recorded in the maxillary sinus puncture procedures.

**Figure 5:**
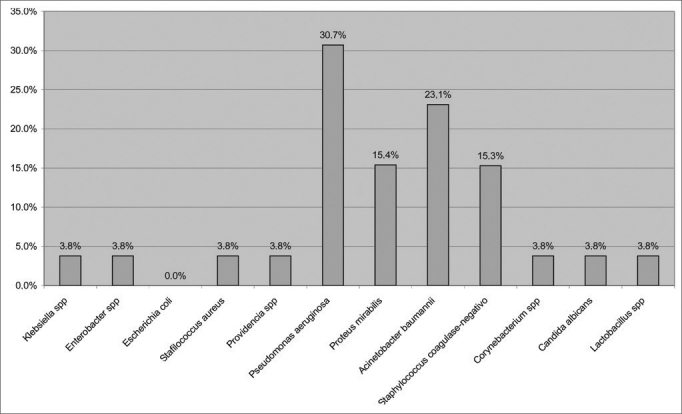
Distribution of microbiological analyses of maxillary sinus aspirates.

Microbiological analysis was done for 26 patients, and at least one agent responsible for IRS was found (Figure 5).

Eight patients (29.6%) underwent FESS. After surgery, seven patients (87.5%) improved from fever and one (12.5%) did not (Table 4). The cause of fever in this patient was attributed to the relapse of a previously diagnosed digestive system tumor.

**Table 4:** Clinical evolution after functional endoscopic sinus surgery.

	No		Yes		*p*-value*
Clinical improvement	n	%	n	%	
	1	12.5	7	87.5	< 0.001

n = number of subjects.

* Test for equality of two proportions.

## DISCUSSION

IRS is one of the causes for fever of unknown origin in ICU patients and, as such, must be thoroughly investigated^2^, ^3^, ^6^, ^7^. This infective syndrome increases patient morbidity and may be associated with difficulty managing some diseases such as VAP.

Prevalence rates were higher among male patients in this study. Head trauma was the main cause for hospitalization in the studied population. These findings were similar to those seen in the literature^9^, ^13^, ^16^, ^24^. This population comprises a group of severe patients who often require prolonged hospitalization. Additionally, they are often on ventilation for longer periods of time (hyperventilation) to control intracranial pressure levels^16^. Consequently, some authors have described lower levels of consciousness, scores under 7 in the Glasgow coma scale, and/or head trauma as risk factor for IRS^25^.

The same imaging criteria described in the literature were used in this study: air-fluid level, complete opacification, or mucosal thickening greater than 6 mm^24^, ^26^, ^27^. In general terms, the most frequently involved paranasal sinuses were the maxillary and the sphenoidal, as seen in 85.2% and 74.1% of the nasal cavities respectively. The ethmoid and frontal sinuses also presented significant rates of involvement. Some authors have described a positive correlation between sinus disease and length of intubation^20^, ^28^, i.e., the longer the period the patient is on the ventilator the greater the number of involved paranasal sinuses. All patients in this study were intubated for over two weeks, which possibly led to the high rates of involvement observed.

One of the most characteristic diagnostic signs of rhinosinusitis is purulent secretion in the middle meatus. Only 30.7% of the nasal cavities of the critical patients enrolled in this study were blocked. Kountakis et al.^13^ e Skoulas et al.^24^ described rates of 25.2% and 40.3% respectively. These low rates indicate that the classic endoscopic signs of rhinosinusitis may be absent in most ICU patients, given the altered levels of immune response of this population^9^, ^10^.

ICU patients with hospital infection were being treated empirically with broad specter antibiotics at the time of their ENT examination. Initial therapy for FUO individuals must consider the endemic hospital bacterial flora. Glycopeptides, carbapenems, and fourth generation cephalosporins were the most frequently used drugs. The drugs were changed by the ICU medical staff and infectologists based on the results of sinus aspirate cultures. There is no consensus in the literature as to the duration of antibacterial administration, but a minimum of seven days of drug therapy seems to be necessary^7^, ^15^.

Some authors believe that antibacterial therapy alone is not enough to treat IRS patients. Studies have shown that ICU patients with IRS still had fever even when the concentration of antibiotic drugs in their sinus aspirates was high enough to attain the minimum inhibitory level to eliminate bacteria. A complex set of pathologic variables can be used to explain this event, among which is the formation of a biofilm in the nasal mucosa preventing antibacterial drugs from reaching their targets. These authors concluded that drainage of the infected secretion is required to treat IRS^20^.

Patients with FUO and radiological signs of rhinosinusitis are advised to remove their nasal devices as part of their initial assessment. Various papers have described nasal devices as one of the main risk factors for the development of IRS^8^, ^14^, ^15^, ^16^. These devices may press against the ostiomeatal complex and mechanically block the sinus ostium. Additionally, trauma produced by this tube may trigger an inflammatory response in the nasal mucosa, mucosal edema, increased secretion, and alterations in the property of mucous fluid^29^.

Other factors have been associated with increased nasal congestion in these individuals: prolonged periods in dorsal decubitus, high central venous pressure, and positive pressure ventilation. These factors increase jugular venous pressure, thus congesting the vessels in the nasal mucosa^29^.

Therefore, patients are required to remove their nasal devices and use nasal topical vasoconstrictors for 72 hours before undergoing invasive procedures^8^, ^17^, ^25^. These measures may reverse the inflammatory process in some individuals and facilitate the drainage of sinus secretions.

Many studies have indicated maxillary sinus puncture as the standard procedure used in to diagnose IRS^8^, ^12^, ^22^, ^26^, ^30^. This procedure may also be a relevant tool in the treatment of critical patients with IRS^8^, ^12^, ^22^, ^26^, ^30^. This study revealed that 70.4% of the patients improved from their fever episodes after the procedure. This outcome is similar to that published by Salord et al.^14^ and Rouby et al.^26^. In the study by Caplan & Hoyt^25^, 58.8% of the patients improved from fever, while Ramadan et al.^15^ published improvement rates of 83%.

No complications were recorded in the maxillary sinus puncture procedure.

Community-acquired rhinosinusitis is usually caused by *Streptococcus pneumoniae and Haemophilus influenzae*. Hospital rhinosinusitis has been related to colonization by endogenous bacteria and pathogens exogenous to the ICU environment^29^. The most frequently microorganisms observed in our study were *Pseudomonas aeruginosa* (30.7%), *Acinetobacter baumannii* (23.1%), *Proteus mirabilis* (15.4%) and coagulase-negative *Staphylococcus* (15.3%). The results of microbiologic analysis indicate that the changes in the nasal and systemic immune profile of these critical patients may enable pathogens to colonize the patients' airways and trigger the onset of IRS. Similar findings were previously reported in the literature^8^, ^21^, ^27^, ^30^, ^31^, ^32^.

Poorly cared for pharynxes, oral cavities and teeth may play an important role in the colonization of paranasal sinuses by bacterial pathogens^29^. Gastric protection and nasoenteral tubes often used in severe patients also favor the spread of bacteria in the stomach^29^.

Previously used nasal devices and the injured mucosa around them may offer a good bed for the development of pathogens. Bacteria populations may grow in these areas and form biofilms. *P. aeruginosas* and *S. aureus* have mucus and respiratory epithelial cell receptors. As *A. baumannii*, they also produce glycocalyx, which adheres to polyvinyl chloride, a material present in endotracheal tubes^7^. In this study, *P. aeruginosas* and *A. baumannii* were the most frequently observed pathogens. Therefore, the presence of biofilm in the nasal mucosa may trigger ongoing inflammatory response and sustained fever episodes even after the infected secretion has been removed from the patients' sinuses. The high rates of involvement of posterior paranasal sinuses, more specifically of the sphenoidal sinus^19^, ^20^, may be a factor in the therapeutical failure of the maxillary sinus puncture procedure^15^. These factors may account for the clinical failure of the puncture procedure in these patients.

In this study, FESS was indicated for patients whose puncture procedures failed to resolve their IRS. In all, 87.5% of the patients improved from fever after FESS. Pádua et al.^19^ also described a high success rate (82%) with surgery.

## CONCLUSION

Maxillary sinus puncture at the bedside enabled the identification of at least one significant pathogen responsible for hospital infective rhinosinusitis. The most frequently observed pathogens were *Pseudomonas aeruginosa* (30.4%) and *Acinetobacter baumannii* (23.1%). The procedure proved to be a relevant therapeutical tool, and showed success rates of 70.4% in managing patient fever.
